# Comparing the effects of team-based and problem-based learning strategies in medical education: a systematic review

**DOI:** 10.1186/s12909-024-05107-9

**Published:** 2024-02-22

**Authors:** Weilin Zhang, Jinsong Wei, Weixiong Guo, Zhongwei Wang, Siyuan Chen

**Affiliations:** Department of orthopaedics, Affiliated Hospital of Guangdong Medical University, Zhanjiang, PR China

**Keywords:** Team-based learning, Problem-based learning, Medical education

## Abstract

**Background:**

Recently, there has been a concerted effort within medical schools to depart from conventional lecture-based learning approaches to alternative teaching methods such as team-based learning (TBL) and problem-based learning (PBL), with the aim of enhancing both student engagement and instructional efficacy. Despite this shift, a comprehensive review that directly compares the impacts of PBL and TBL methods in medical education is lacking. This study seeks to address this gap by conducting a meta-analysis that compares the effects of TBL and PBL in the context of medical education.

**Methods:**

Studies from Embase, PubMed, Web of Science, China National Knowledge Infrastructure, and Chinese Wanfang Database were searched, from inception to July 11, 2023. A meta-analysis was performed using Stata 14.0, and a total of 10 studies (including 752 participants) were included. The standardized mean difference (SMD) was used to estimate pooled effects. Heterogeneity was detected using the I2 statistic and further explored using meta-regression analysis.

**Results:**

Compared with PBL, TBL significantly increased the number of theoretical tests (SMD = 0.37, 95% CI: 0.02–0.73). Additionally, TBL greatly improved teamwork skills compared with PBL. However, there were no significant differences between the TBL and PBL groups concerning practical skill scores, learning interest, or understanding skills.

**Conclusion:**

TBL in the theoretical aspects of medical education appears to be more effective than PBL in improving theoretical test scores and teamwork skills, providing evidence for the implementation of TBL in medical education.

**Supplementary Information:**

The online version contains supplementary material available at 10.1186/s12909-024-05107-9.

## Background

Medical education is constantly evolving to meet changing needs and expectations. One of the major challenges faced by medical educators is designing and implementing effective teaching and learning methods that can foster students' knowledge, skills, attitudes, and behaviors for future professional practice [1, 2]. Particularly, two student-centered methods have received considerable attention: team-based learning (TBL) and problem-based learning (PBL).

PBL is a problem-solving learning method that has many advantages such as the following: facilitating knowledge acquisition and retention; stimulating problem-solving ability; enhancing intrinsic learning interest; deepening learning; improving communication, teamwork, presentation, and critical evaluation skills; fostering self-directed learning ability; and strengthening clinical skills [3, 4]. However, some people are concerned about the achievement of PBL in basic science knowledge compared to traditional methods because PBL students tend to score lower on basic science knowledge tests [5, 6].

TBL is a relatively new teaching method, and it has become increasingly popular in medical education over the past decade. TBL is similar to PBL and integrates active learning strategies into preclinical medical courses [7]. Both PBL and TBL use collaborative learning methods to promote critical thinking and team-building skills, which are essential for medical students’ future career development [8]. Furthermore, they are learner-centered teaching methods, encouraging students to work together to solve problems related to their profession. Both teaching methods ensure that learners use these problems to build and apply existing knowledge [9].

Although both TBL and PBL are active and collaborative problem-solving methods, they differ in several aspects such as the sequence of learning activities, size and composition of groups, nature and format of problems, the role of teachers, and evaluation methods. These differences may affect the students’ learning outcomes and experiences. Therefore, it is necessary to compare and synthesize the effects of TBL and PBL in medical education. Therefore, this study compares the effects of TBL and PBL in medical education through a meta-analysis.

## Methods

This meta-analysis followed the guidelines of the Preferred Reporting Items for Systematic Reviews and Meta-Analyses (PRISMA) statement [10].

### Search strategy

Two independent reviewers performed the literature search. The databases searched included Embase, PubMed, Web of Science, China National Knowledge Infrastructure, and Chinese Wanfang Database. The search terms used included the following: “TBL,” “Team-based learning,” “PBL,” and “problem-based learning.” The search was completed on July 11, 2023. Details of the search strategy are provided in Additional file 1.

### Selection criteria

Studies that met the following criteria were included: 1) randomized controlled studies; 2) either TBL or PBL was received as an educational intervention by participants who were healthcare professionals or medical students; and 3) at least one of theoretical test scores, practical ability, and questionnaire surveys was measured as an outcome.

The exclusion criteria were as follows: (a) studies that were non-randomized and non-controlled; (b) studies with partial data duplication; and (c) conference abstracts and review articles.

### Data extraction and quality assessment

Two reviewers independently extracted data from the eligible studies. Discrepancies were resolved via discussion and consensus. The extracted data included the name of the first author, year of publication, number of participants in the intervention and control groups, study specialty, and outcome measures.

The quality of each included study was independently assessed by two reviewers using the risk of bias table according to the Cochrane Collaboration [11]. This tool has seven domains: sequence generation, allocation concealment, blinding of participants and personnel, blinding of outcome assessments, incomplete outcome data, selective outcome reporting, and other sources of bias. Each domain received a rating of “low risk,” “high risk” or “unclear risk.” A study scored “low risk” in overall bias if all domains were rated as “low risk,” “some concern” if any domain received a “some concern” rating, and “high risk” if at least one domain had a “high risk” rating or several domains had a “some concern” rating. Disagreements were resolved through discussion to achieve a consensus.

### Statistical analysis

The data were analyzed using Stata version 14.0. The data were presented as weighted mean differences (WMD) and 95% confidence intervals (CIs). The impact of heterogeneity on the results was evaluated using the I-squared (I^2^) test. According to the Cochrane review guidelines, the fixed-effects model was employed to pool data if there was no heterogeneity (I^2^ < 50%); otherwise, the random-effects model was adopted when severe heterogeneity was present at I^2^ > 50% (or the value of I^2^ was close to 50%). If there was significant heterogeneity between studies, a meta-regression analysis was used to further explore the sources of heterogeneity. A sensitivity analysis was performed to investigate the influence of a single study on the overall pooled estimate by the sequential deletion of each study. Publication bias was evaluated using Egger’s test.

## Results

### Literature search results

**Fig. 1:**
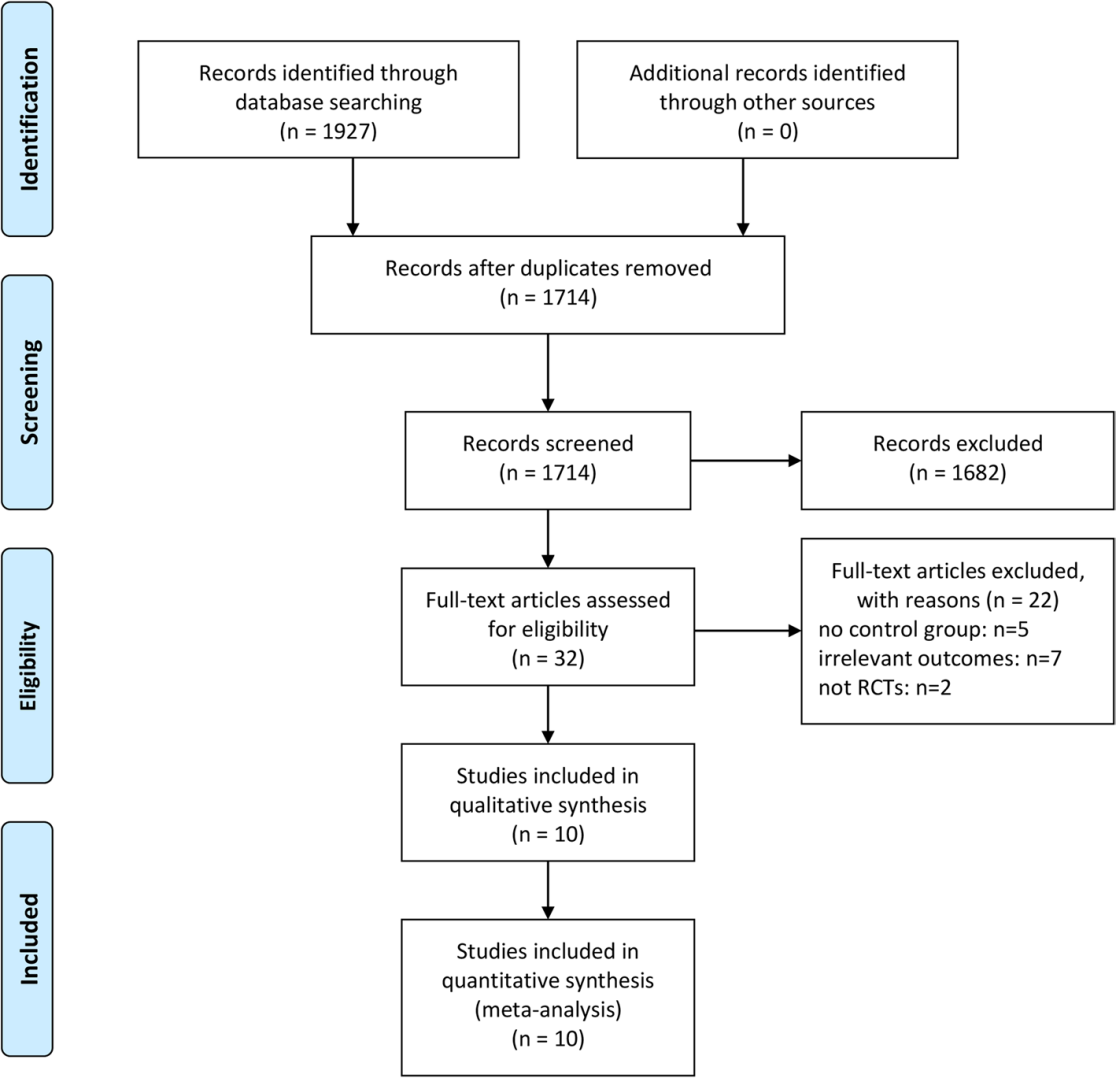
Flowchart of the selection process of the included studies

The databases were searched and 1927 records were identified. After 213 duplicates were removed, the titles and abstracts of 1714 articles were screened and 1682 that did not meet the inclusion criteria were excluded. The full texts of 32 articles were then assessed, and 22 were excluded for the following reasons: 5 had no control group, 7 had irrelevant outcomes, and 10 were not randomized controlled trials. Finally, ten studies were included in the meta-analysis [12–21]. A flow diagram of the selection process is shown in Fig. 1.

### Basic characteristics of enrolled studies

Publication dates of the included studies ranged from 2014 to 2023. The TBL and PBL groups had sample sizes of 12–115 and 12–85 residents, respectively, with a total of 752 participants (TBL group: 391; PBL group: 361). The studies spanned nine specialties: neurology, oncology, clinical medicine, ophthalmology, general surgery, acupuncture, gynecology and obstetrics, urology of Chinese medicine, and stomatology. All the studies used test scores as the outcome measure to compare the effectiveness of the two pedagogical methods. Table 1 summarizes the main characteristics of the included studies.

**Table 1:** Characteristics of included studies

Study	Disciplines or curricula	Sample size (TBL/PBL)	Participant characteristics	Outcome assessment
Vakani 2014 [18]	Neurology	59 (30/29)	Physicians	Theoretical tests scores, questionnaire surveys
Dong 2015 [12]	Oncology	41 (20/21)	Oncology graduate students	Theoretical tests scores
Yang 2016	Clinical medicine	164 (82/82)	Students of clinical medicine	Theoretical tests scores
Han 2017 [14]	Ophthalmology	60 (30/30)	Resident doctors	Theoretical tests scores
He 2017 [15]	General surgery	200 (115/85)	Clinical medical students	Theoretical tests scores, Practical ability
Gong 2017	Acupuncture discipline	80 (40/40)	Graduate trainees in acupuncture and moxibustion	Theoretical tests scores
Chen 2018	Ophthalmology	24 (12/12)	Ophthalmology residents	Theoretical tests scores, Practical ability, questionnaire surveys
Wang 2020	Gynaecology and obstetrics	36 (18/18)	Obstetrics and gynecology intern	Theoretical tests scores
Zhao 2022	Urology of Chinese medicine	64 (32/32)	Chinese medicine residents	Theoretical tests scores, questionnaire surveys
Xie 2023 [19]	Stomatology	24 (12/12)	Residents in stomatology	Theoretical tests scores, Practical ability, questionnaire surveys

### Quality assessment

**Fig. 2:**
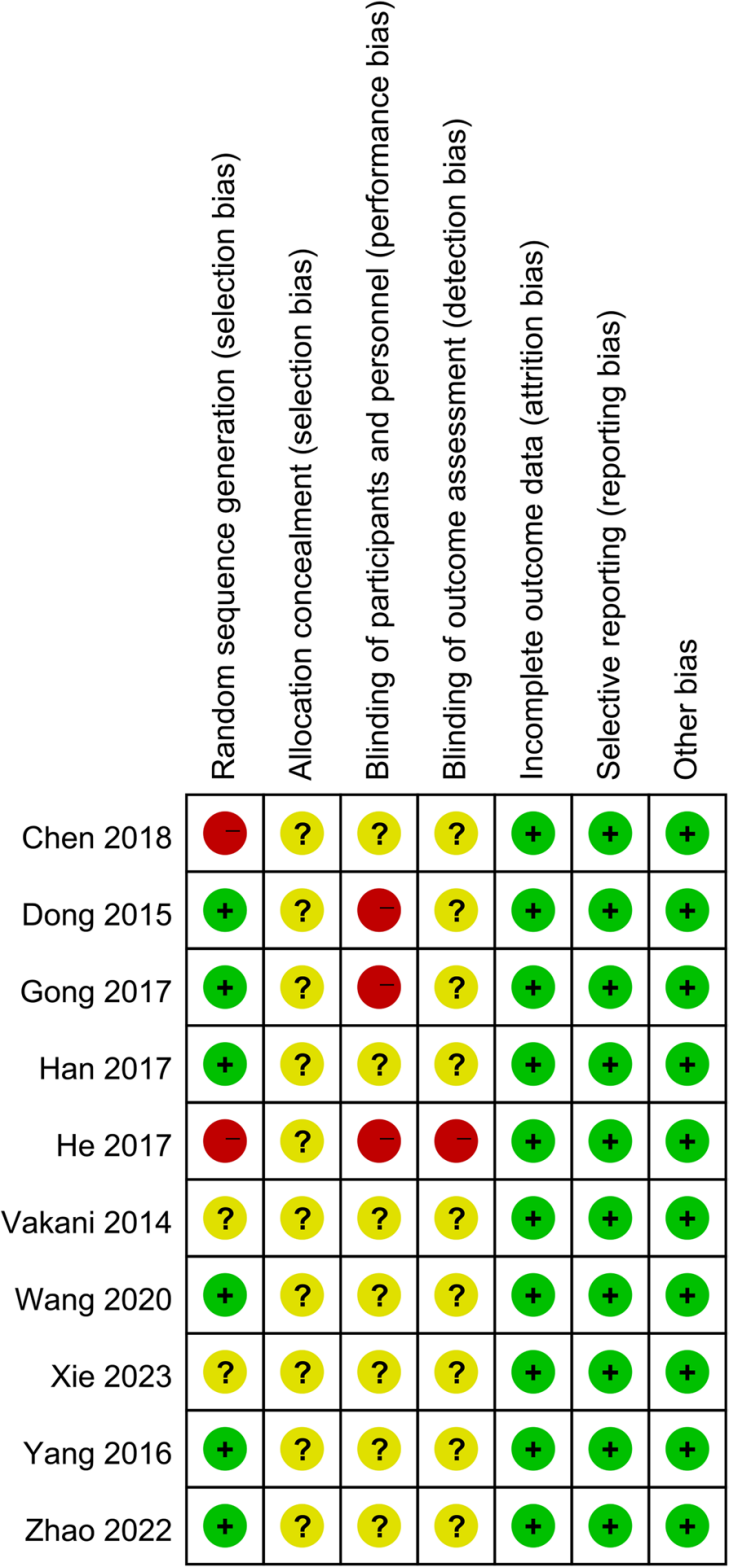
Risk of bias summary for each included study

The methodological quality of the included studies is shown in Fig. 2. Most of the studies used appropriate randomized sequence generation methods. All the studies were free of selective reporting or other biases. Allocation concealment or blinding were not present.

### Data synthesis

#### Theoretical test scores

**Fig. 3:**
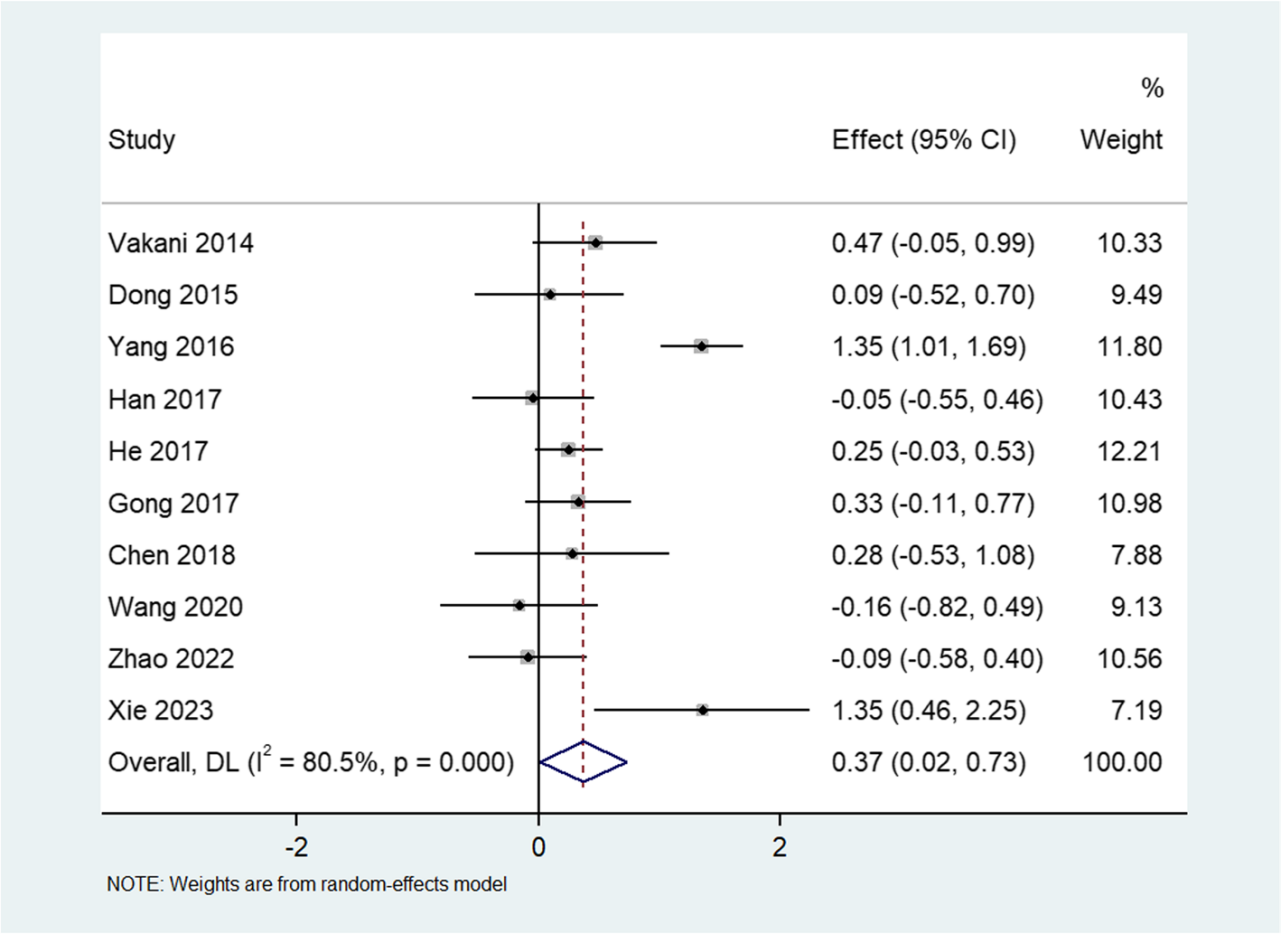
Forest plot for the effects of TBL on theoretical test scores compared with PBL

Ten articles involving 944 participants reported theoretical test scores. There was high heterogeneity among the studies (I^2^ = 80.5%, *P* < 0.0001); therefore, a random effects model was used. The meta-analysis showed that the theoretical test scores were significantly higher in the TBL group than in the PBL group (standardized mean difference [SMD] = 0.37, 95% CI: 0.02–0.73) (Fig. 3).

**Fig. 4:**
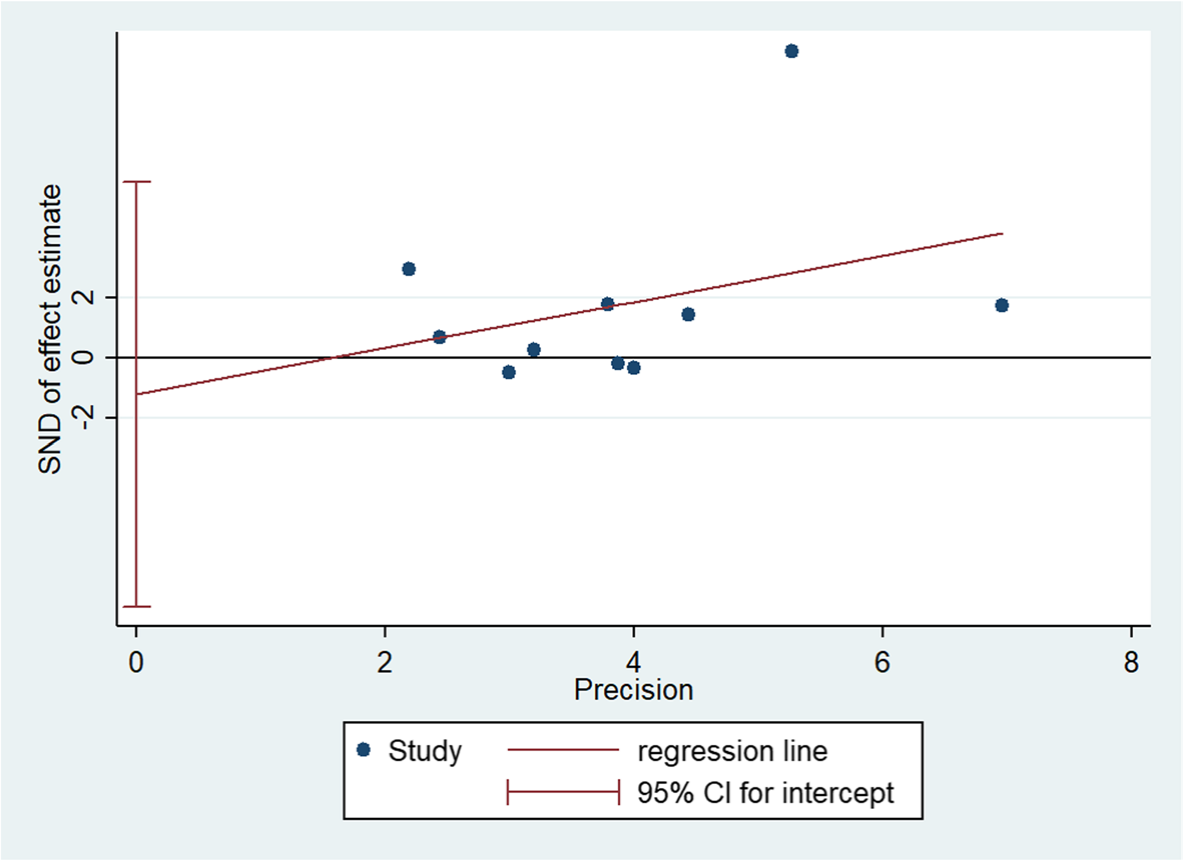
Egger’s test of theoretical test scores for publication bias assessment of all included studies

To identify the sources of heterogeneity among the studies, we performed a regression analysis based on four covariate factors: discipline, country, year, and identity (student or doctor). The regression coefficients of these factors were not statistically significant (*P* > 0.05), indicating that they did not affect the pooled SMDs (Table 2). Owing to the significant heterogeneity among the studies, we conducted a sensitivity analysis by sequentially excluding each study to re-evaluate the quality and consistency of the results. However, the source of heterogeneity could not be clearly attributed to any single study. Additionally, Egger’s test showed no publication bias (*P*=0.606), indicating the reliability of the results (Fig. 4).

**Table 2:** Meta-regression analysis of included studies for exploration of the sources of heterogeneity

Factors	Coefficient	Standard error	95% Confidence interval	*P*
Disciplines	0.5787264	0.5137159	-0.4281382 - 1.585591	0.26
Country	0.5002787	1.474001	-2.38871 - 3.389267	0.734
Year	-13.09959	177.2125	-421.7524 - 395.5533	0.943
Identities	0.1852473	0.737756	-1.516021 - 1.886516	0.808

#### Practical skill scores

**Fig. 5:**
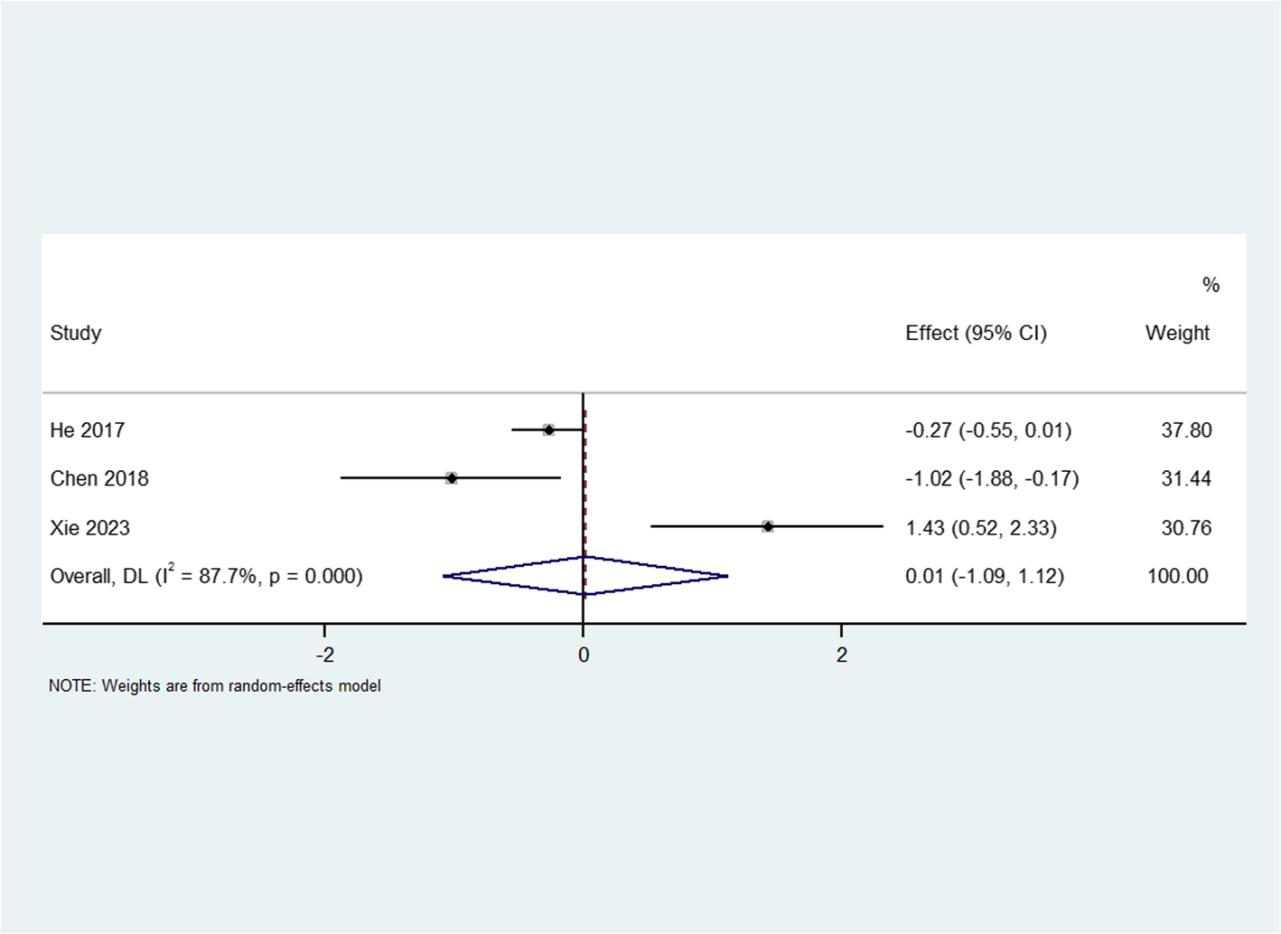
Forest plot for the effects of TBL interventions on practical skill scores compared with PBL

**Fig. 6:**
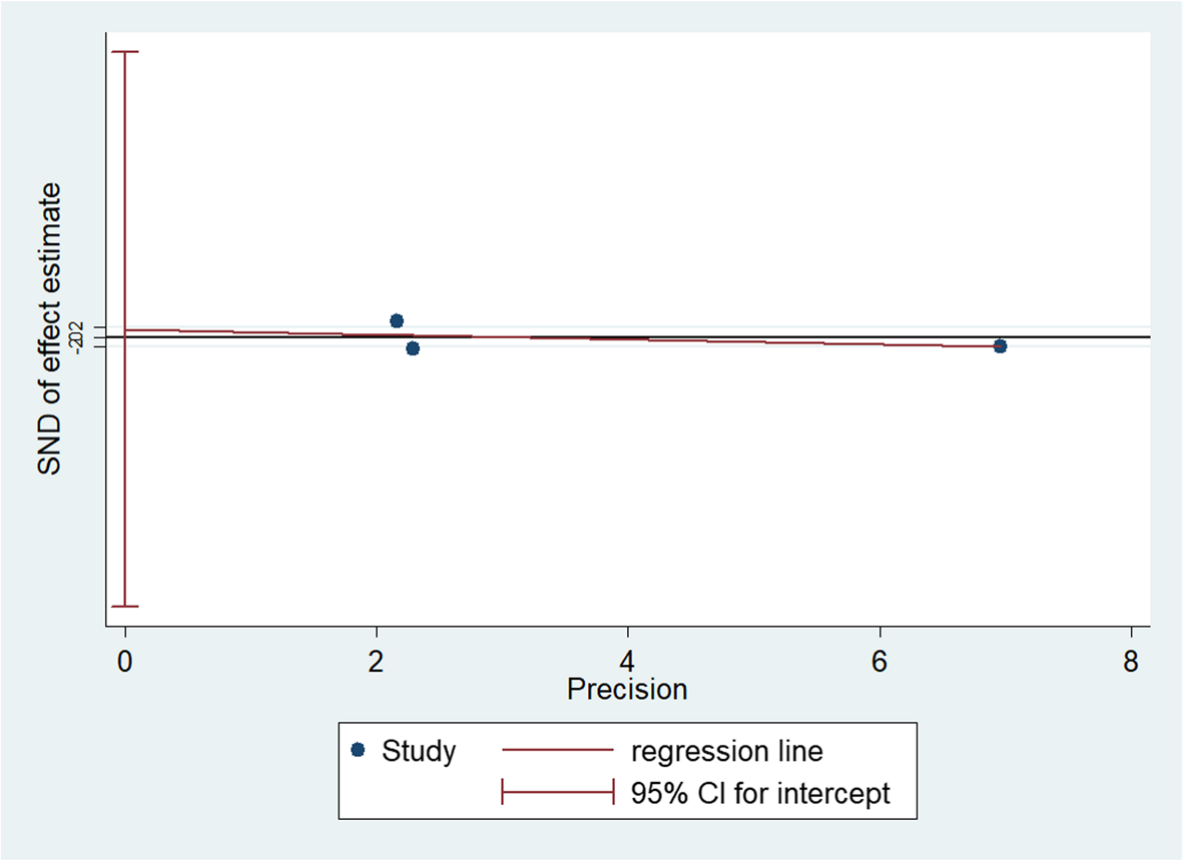
Egger’s test of practical skill scores for publication bias assessment of all included studies

Practical skill scores were pooled from three articles with 248 participants. Significant heterogeneity existed among the studies (I^2^ = 87.7%, *P* < 0.0001); therefore, a random-effects model was used for the analysis. The pooled results showed that the practical skill scores were not significantly different between the TBL and PBL groups (SMD = 0.01, 95% CI: -1.09–1.12) (Fig. 5). Owing to the significant heterogeneity between the studies, we performed a sensitivity analysis. The source of heterogeneity found by excluding each study could not be clearly attributed to any one study. Additionally, Egger’s test showed no publication bias (*P*=0.35), indicating that the results were reliable (Fig. 6).

#### Various qualities and abilities

**Fig. 7:**
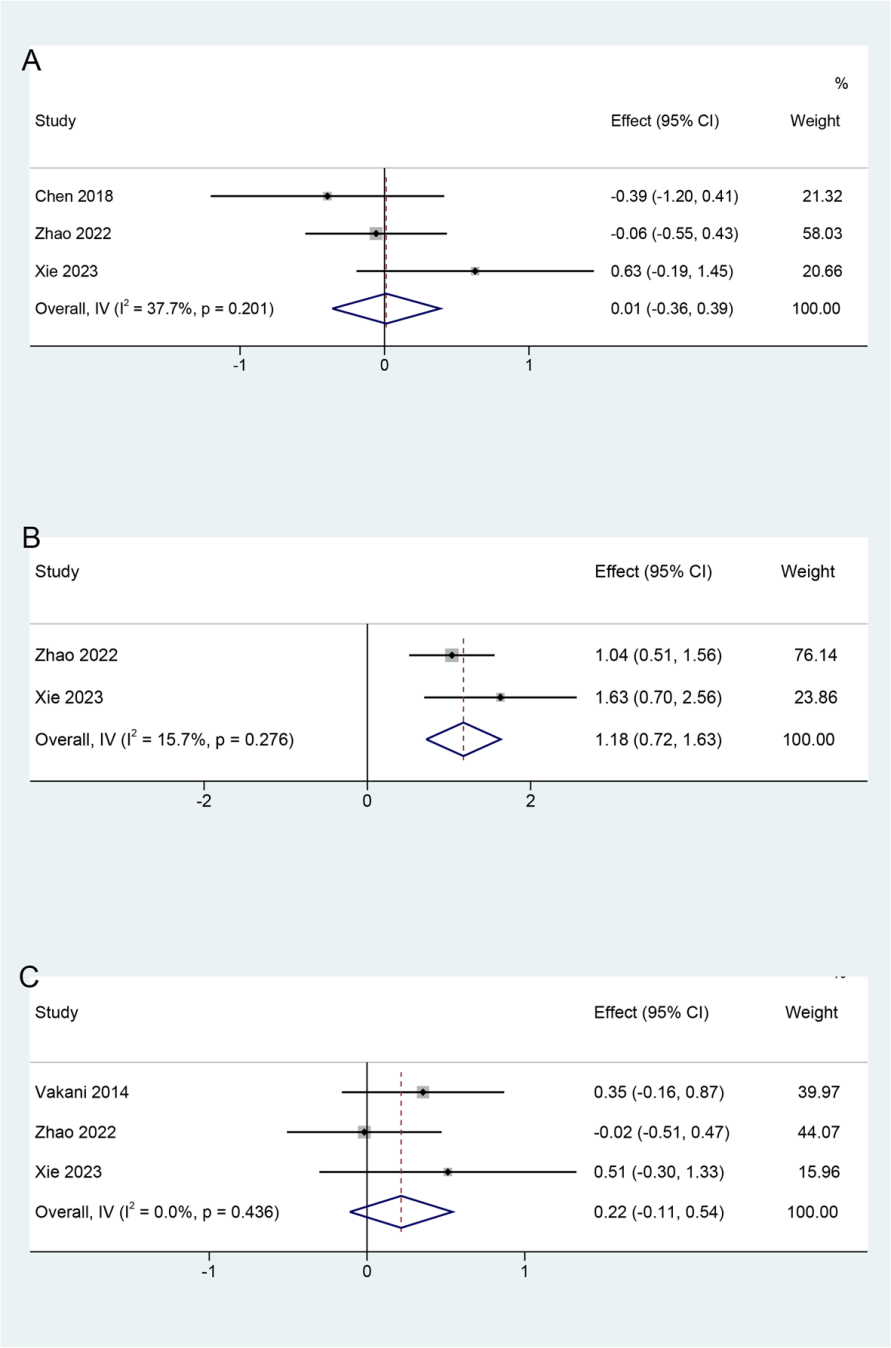
Forest plot of students’ various qualities and abilities for TBL compared with PBL. **a** Effects on developing learning interest; **b** Effects on developing teamwork skills; **c** Effects on developing understanding skills

Three studies assessed learning interest. Heterogeneity was not found among the study results (I^2^ = 37.7%, *P* = 0.201); therefore, a fixed-effects model was used. The results showed that there was no significant difference between the TBL and PBL groups in improving learning interest (SMD = 0.01, 95% CI: -0.36–0.39) (Fig. 7A).

Two studies assessed teamwork skills. No significant heterogeneity was found between the studies (I^2^ = 15.7%, *P* = 0.276); therefore, a fixed-effects model was used. The results demonstrated that TBL can significantly improve teamwork skills compared with PBL (SMD = 1.18, 95% CI: 0.72–1.63) (Fig. 7B).

Three studies assessed the students’ understanding skills. There was no heterogeneity among the studies (I^2^ = 0%, *P* = 0.436); therefore, a fixed-effects model was used. The pooled effect size showed no significant difference in understanding skills (SMD = 0.22, 95% CI: -0.11–0.54) in favor of TBL, compared with PBL (Fig. 7C).

## Discussion

PBL is implemented through small-group tutorials, typically comprising 8–10 students guided by a tutor. These sessions aim to identify and analyze a problem or scenario, delineate essential concepts, generate and deliberate ideas, and establish primary learning objectives [22]. The participants were expected to investigate these topics independently and exchange their findings at subsequent gatherings. Conversely, TBL employs a student-centered instructional approach designed for larger classes [23]. Students are usually divided into small teams of five to seven members and work together to solve clinically relevant problems [24]. Both PBL and TBL utilize professionally relevant problems and small-group learning but differ in terms of facilitation and structure [9]. In TBL, one teacher facilitates the interactions among multiple self-managed teams [9]. Additionally, TBL incorporates mandatory pre-reading assignments and tests of prior knowledge, whereas PBL focuses on activating prior knowledge and encouraging students to identify concepts they struggle with for further study [25]. TBL also includes inter-team discussions, structured feedbacks, and problems with related questions [26–28]. To compare the effects of TBL and PBL in medical education, this study conducted a meta-analysis of ten randomized controlled trials.

This study revealed that the TBL group achieved significantly higher scores on the theoretical test than the PBL group, suggesting that TBL can effectively enhance the knowledge level of medical students. This discovery validates previous concerns [29, 30]. Deliberate preparation for indispensable knowledge acquisition in the context of TBL was devised to shift the onus of content assimilation during class [31]. Nonetheless, students’ compliance with the assigned pre-reading and preparation fell short of their expectations. Deficiency in preparation adversely affects team learning and performance of teams [30]. Furthermore, one study discovered that students rated TBL as the least efficacious pedagogical approach, with only 11% favoring it compared to 21% for PBL, 29% for lectures, and 39% for self-directed learning [29]. Additionally, the findings of our study are consistent with those of previous studies. Burgess et al. ascertained that TBL, as a substitute for PBL in the first and second years of the medical curriculum, furnished a standardized framework for small-group learning on a large scale while also yielding resource efficiency [32]. This may be associated with TBL emphasizing knowledge mastery and review. TBL ensures students’ comprehension and retention of basic knowledge through pre-class preparation and in-class quizzes, while also enhancing their application and extension of knowledge through group discussions and teacher feedback [33, 34]. Although PBL can also promote students’ exploration and discovery of knowledge, it may result in inadequate and superficial mastery of knowledge owing to a lack of systematization and standardization [35].

This study found that TBL can significantly enhance teamwork skills compared with PBL. However, the two teaching methods did not have significant differences in their effects on practical skills, learning interests, or understanding skills. This study has some consistencies and differences from previous studies. For example, Hopper et al. pointed out that both TBL and PBL are task-based learning strategies, but they have different goals, processes, and assessment methods [36]. TBL emphasizes achieving language learning objectives by completing specific tasks, whereas PBL emphasizes developing critical thinking, problem-solving, and creativity skills by solving complex real-world problems. Hopper et al. pointed out that both TBL and PBL can promote group collaboration, but TBL is more suitable in the language teaching field [36]. In contrast, Burgess et al. conducted an experiment in medical education that used TBL instead of PBL. The experiment found that after using TBL, hospital nurses’ teamwork skills significantly improved and they cared more about the patients they encountered [32]. These results suggest that, in the medical field, TBL may have better effects than PBL.

This study has some limitations. First, the studies included covered multiple disciplines, which may have introduced heterogeneity. Although we conducted regression and sensitivity analyses to explore the sources of heterogeneity, we did not find any significant factors. However, we still cannot completely rule out the possibility of other potential confounding factors. Second, the evaluation indicators used in the included studies may have some subjectivity and bias. For example, theoretical exam scores may be affected by factors such as the difficulty of the questions, scoring criteria, and level of the examinees. Furthermore, the evaluation of practical skills, learning interest, teamwork skills, and comprehension ability may be affected by factors such as questionnaire design, rater subjectivity, and evaluation timing. Finally, the specific implementation methods of TBL and PBL used in the studies included in this review may have some variations. For example, course content, duration, frequency, group size, teacher role, and learning resources of TBL and PBL may differ. Therefore, future studies should clearly describe the specific operational details of TBL and PBL and use more objective and standardized evaluation indicators to improve the credibility of the results.

## Conclusions

In summary, this study compared the effects of TBL and PBL teaching methods in medical education through a meta-analysis and found that TBL can improve medical students’ theoretical test scores and teamwork skills. However, there were no obvious advantages in enhancing practical skills, learning interest, and understanding skills. These results provide useful references for medical educators, but they also need to be applied flexibly according to the actual situation. Future studies should further explore the applicability and optimization strategies of TBL and PBL considering different professional fields, teaching objectives, and teaching environments with the aim of contributing to the improvement of medical education quality.

### Supplementary Information

**Additional file 1.** The details of the search strategy.

## Data Availability

The datasets used and/or analysed during the current study available from the corresponding author on reasonable request.

## Abbreviations

TBL: Team-based learning
PBL: Problem-based learning
WMD: Weighted mean differences
CIs: Confidence intervals
I^2^: I-squared
SMD: Standardized mean difference
