# Supplementary material for: Comparing the effects of team-based and problem-based learning strategies in medical education: a systematic review
Source: BMC Med Educ. 2024 Feb 22;24:172. doi: 10.1186/s12909-024-05107-9 (PMC10885648; doi:10.1186/s12909-024-05107-9)
Supplement: Supplementary file 1 — Additional file 1. The details of the search strategy. [file 12909_2024_5107_MOESM1_ESM.docx]

Additional file 1. The details of the search strategy.

Pubmed

((Team-based learning[Title/Abstract]) OR (TBL[Title/Abstract])) AND ((Problem based learning[Title/Abstract]) OR (PBL[Title/Abstract]))

Web of science

(((Team-based learning) OR (TBL)) AND ((Problem based learning) OR (PBL)))

Embase

#1 ‘Team-based learning’ OR ‘TBL’

#2 ‘Problem based learning’ OR ‘PBL’

#3 #1 AND #2
